# Diversity, Distribution and Quantification of Antibiotic Resistance Genes in Goat and Lamb Slaughterhouse Surfaces and Meat Products

**DOI:** 10.1371/journal.pone.0114252

**Published:** 2014-12-05

**Authors:** Leyre Lavilla Lerma, Nabil Benomar, Charles W. Knapp, David Correa Galeote, Antonio Gálvez, Hikmate Abriouel

**Affiliations:** 1 Área de Microbiología, Departamento de Ciencias de la Salud, Facultad de Ciencias Experimentales, Universidad de Jaén, 23071, Jaén, Spain; 2 Department of Civil and Environmental Engineering, University of Strathclyde, Glasgow, Scotland, United Kingdom; 3 Departamento de Microbiología del Suelo y Sistemas Simbióticos, Estación Experimental del Zaidín, Agencia CSIC, Granada, Spain; University Hospital of the Albert-Ludwigs-University Freiburg, Germany

## Abstract

The distribution and quantification of tetracycline, sulfonamide and beta-lactam resistance genes were assessed in slaughterhouse zones throughout meat chain production and the meat products; this study represents the first to report quantitatively monitor antibiotic resistance genes (ARG) in goat and lamb slaughterhouse using a culture independent approach, since most studies focused on individual bacterial species and their specific resistance types. Quantitative PCR (qPCR) revealed a high prevalence of tetracycline resistance genes *tetA* and *tetB* in almost all slaughterhouse zones. Sulfonamide resistance genes were largely distributed, while beta-lactam resistance genes were less predominant. Statistical analysis revealed that resistant bacteria, in most cases, were spread by the same route in almost all slaughterhouse zones, except for *tetB*, *bla_CTX_* and *bla_TEM_* genes, which occurred in few zones as isolated ‘hot spots.’ The sum of all analyzed ARG indicated that slaughterhouse surfaces and end products act as reservoirs of ARG, mainly *tet* genes, which were more prevalent in slaughtering room (SR), cutting room (CR) and commercial meat products (MP). Resistance gene patterns suggest they were disseminated throughout slaughterhouse zones being also detected in commercial meat products, with significant correlations between different sampling zones/end products and total resistance in SR, CR and white room (WR) zones, and also refrigerator 4 (F4) and MP were observed. Strategically controlling key zones in slaughterhouse (SR, CR and WR) by adequate disinfection methods could strategically reduce the risks of ARG transmission and minimize the issues of food safety and environment contamination.

## Introduction

Antibiotics have been routinely used for therapy, prophylaxis, animal growth promotion and in agricultural operations for several decades. However, the over- or in-appropriate use results in the selection of drug-resistant pathogens and commensals in animals and the environment [1], with resistant microorganisms spreading through water and food chain. As such, the prevalence and distribution of antibiotic-resistant bacteria (ARB) have become a threat to food safety; the surveillance and control of spread of antibiotic resistance genes (ARG) throughout food chain has great relevance since consumers are increasingly aware of concerns over antibiotic resistant bacteria in foods, especially those of animal origin. Furthermore, several studies unequivocally supported the concern that use of antibiotics in veterinary or in food animals (particularly non-therapeutic use) impacts the health of people on farms and within the food chain [2]–[7].

There is a growing interest in ecological studies of antimicrobial resistance in foodborne bacteria. Those bacteria are considered potential reservoirs of resistance as a consequence of the complex transmission routes between farms and consumers. The frequent transfer of resistance genes among host bacteria is becoming more evident with molecular studies, which have shown the distribution of the same gene in different bacteria of animal or human origin [7]. For example, the spread of ARG from animals to humans could be enhanced within the food matrix and also within the human gastrointestinal tract [8]–[10] by horizontal gene transfer of mobile elements such as plasmids, transposons, integrons or phages [11]–[13]. In fact, serious public health hazards arise because of the ability of many bacteria to acquire resistance traits to different antimicrobials.

Smith DeWaal and Vaughn Grooters [14] report that there has been a significant increase in sales and distribution of the highly important classes of antibiotics (tetracyclines, beta-lactams and sulfonamides) frequently used for therapeutic and prophylactic purposes in food-producing animals. A recent increase in antibiotic-resistant foodborne outbreaks highlights the emergence of resistance [14]. However, the information available on the incidence of resistance in foodborne bacteria is mainly based on phenotypic tests and culture-dependent methods; quantification of ARG in food samples by culture-independent methods should also be used to reveal if there is any real increase in resistance potential. The main goal of the present study was to quantitatively track the frequency and the distribution of ARG in different slaughterhouse surfaces throughout meat chain production (and in the commercial meat products) by quantitative real-time PCR for tetracycline, beta-lactam and sulfonamide resistance genes. Furthermore, the present study determines whether relationships exist between different ARG, and their source locations.

## Material and Methods

### Samples

The samples were collected from a local goat and lamb slaughterhouse that is representative of the region (Jaén, Spain), as described in a previous study by Lavilla Lerma et al. (2013). Standard cleaning and disinfection procedures were applied to sampling areas 12 h before the sampling. Briefly, different samples were collected with sterile swabs from 100-cm^2^ surfaces in the following zones: entrance (E), slaughtering-room (SR), refrigerator (F), cutting-room (CR), freezing-tunnel (FrT) and white-room (WR, where meat products were packaged under controlled environmental conditions). The samples were transported at 4°C to the laboratory, where sterile swabs were then immersed in 10 ml of sterile BHI (Brain Heart Infusion, Scharlab, Barcelona, Spain) broth and incubated at 22°C for 24 h [15]. There was no observed growth resulting from the incubation. For the present study, 1 ml of samples (each one in duplicate) was centrifuged and the pellets were subjected to DNA extraction. In addition to slaughterhouse surfaces, we examined five meat products (MP; MP1, minced beef; MP2-MP4, ham; MP5, cooked ham) from different supermarkets in Jaén (Spain). Meat product samples (5 g each) were diluted in 45 ml of sterile saline solution (0.85%), homogenized for 3 min in a Stomacher 80 (Biomaster) and then 1 ml of this mixture was added to 9 ml BHI and incubated at 22°C for 4 h. The samples were processed, as described above, for slaughterhouse samples.

### DNA extraction

Total DNA was extracted from the pellets of all samples analyzed in the present study by the method described by De los Reyes-Gavilan et al. [16]. Quality of DNA samples was checked spectrophotometrically and then diluted 1/10 with molecular biology grade water. The integrity of nucleic acids was assessed by electrophoresis of 2 µl of each sample through a 1.2% agarose-TBE gel as described by Sambrook et al. [17].

### Real-time PCR assays for quantification of tetracycline, beta-lactam and sulfonamide resistance genes

The distribution of nine gene determinants targeting tetracycline resistance (*tet*), extended-spectrum beta-lactamases (*bla*), and sulfonamide resistance (*sul*) were selected on the basis of the reported incremented resistance to such antibiotics in foodborne bacteria. In particular, the following gene determinants were assayed: two beta-lactam resistance genes (*bla_TEM_* and *bla_CTX_*), four tetracycline resistance gene determinants (*tet*A, *tetB, tetO* and *tetQ*) and three determinants of sulfonamide resistance (*sulI*, *sulII* and *sulIII*), and *pheS* (phenylalanyl-tRNA synthase) gene universally present in all bacteria as a surrogate measure of bacterial abundance. These genetic markers were selected as bio-indications of relative, potential risks of ARB contamination in slaughterhouse. For each resistance determinant, duplicate 25 µl reaction mixtures including 3.75 µl of DNA template (1/10), 1.25 µl of primer mixture at 10 mM (for each forward and reverse primer, Table 1), 12.5 µl of 2x qPCR Master Mix with SYBR Green for the BioRad iCycler (Primer design Ltd, Southampton, United Kingdom) and 7.5 µl molecular-grade water were analyzed. Analyses were performed using a BioRad iQ5 Real Time PCR Detection System and software (BioRad, Hercules, CA). Reaction conditions included initial denaturation at 95°C (1 min 30 sec for all resistance genes and 2 min for *pheS* gene), and then 40 cycles of 95°C (30 sec) for *tet* and *sul* genes, 50 cycles of 95°C (30 sec) for *bla_TEM_* and 45 cycles of 95°C (30 sec) for *bla_CTX_* gene and 35 cycles of 95°C (1 min) for *pheS* gene, annealing temperatures (X°C, Table 1) for 30 sec, and 72°C for 30 sec for all genes except *pheS* (for 35 sec).

**Table 1:** Primers and conditions used in this study.

Target	Primer	Sequence (5′-3′)	Annealing temperature (°C)	Reference
*pheS*	pheS 21-F	CAYCCNGCHCGYGAYATGC	46	[19]
	pheS 23-R	GGRTGRACCATVCCNGCHCC		
*tet*(A)	TetA-F	GCTACATCCTGCTTGCCTTC	55	[43]
	TetA-R	CATAGATCGCCGTGAAGAGG		
*tet*(B)	TetB-F	TTGGTTAGGGGCAAGTTTTG	55	[43]
	TetB-R	GTAATGGGCCAATAACACCG		
*tet*(O)	TetO-F	AACTTAGGCATTCTGGCTCAC	55	[43]
	TetO-R	TCCCACTGTTCCATATCGTCA		
*tet*(Q)	TetQ-F	TTATACTTCCTCCGGCATCG	55	[43]
	TetQ-R	ATCGGTTCGAGAATGTCCAC		
*sulI*	SulI- F	CGCACCGGAAACATCGCTGCAC	65	[37]
	SulI- R	TGAAGTTCCGCCGCAAGGCTCG		
*sul II*	SulII- F	TCCGGTGGAGGCCGGTATCTGG	57.5	[37]
	SulII- R	CGGGAATGCCATCTGCCTTGAG		
*sul III*	SulIII- F	TCCGTTCAGCGAATTGGTGCAG	61	[37]
	SulIII- R	TTCGTTCACGCCTTACACCAGC		
*bla*_CTX_	CTX-consensus primer F	GCAGYACCAGTA ARGTKATGGC	58	Modified from [44]
	CTX consensus primer R	ATCACKCGGRTCGCCXGGRAT		
*bla*_TEM_	BlaTEM- F	TCGGGGAAATGTGCG	50	[38]
	BlaTEM- R	GGAATAAGGGCGACA		

All reactions were run with serially diluted specific standards of known quantity for each gene as described elsewhere (Table 1) including negative control in each run. The standard curve was generated by cloning gene segments into a plasmid vector (TOPO-TA, Invitrogen-Life Technologies). Plasmids were purified (Plasmid Mini Kit, Qiagen) and the DNA quantified with a UV spectrophotometer (NanoDrop 1000; Thermo Scientific, United Kingdom), and serially diluted to generate concentrations for standard curve [18]. Correlation coefficients (r^2^) for the standards curves were >0.99 for calibration curves, the efficiency varied from 95 to 103% and *log* gene abundance values were always within the linear range of detection. Antibiotic resistance gene (ARG) abundances were normalized to *pheS* housekeeping gene abundances (a surrogate measure of ‘total bacteria’) [19] to minimize variance caused by differential extraction and analytical efficiencies, and differences in background bacterial abundances. These normalized values were then *log*-transformed to apply the Kolmogorov-Smirnov test for normality.

### Statistical analysis

All statistics were conducted using IBM SPSS Statistics version 19. Values of ARG *log*-transformed among the studied zones were compared to determine significant differences between them by a Tukey or a Games Howell test, depending to the Levene test value. The ARG values for a given zone that had a *p*-value of <0.05 were considered statistically significantly different.

To investigate the relationship between relative abundances in different zones or different genes, Pearson correlation coefficients (*r*) were calculated; this determined the extent to which values of two variables (zones or genes) were linearly similar. Categorization of correlations between different antimicrobials was based on Dancey and Reidy [20]; the strength of correlation was considered ‘strong’ when *r*>0.7, moderate when 0.4<*r*<0.6, and weak with *r*<0.3. In all analyses, a *P* value of <0.05 was considered significant for two-tailed tests.

## Results and Discussion

### Spatial variations of antibiotic resistance gene abundances in goat and lamb slaughterhouse

Slaughterhouses comprise several zones where meat and product processing occur, and each zone has characteristic environmental conditions and surface exposures that may influence bacteria presence and retention. The flow of ARB and their ARG throughout meat chain production has been documented in the literature, mainly in poultry and swine slaughterhouses [21]–[23], and many studies have focused on individual bacteria species and specific resistance traits [24], [25]. To our knowledge, this is the first report of the monitoring of antibiotic resistance in the total microbiota present in goat and lamb slaughterhouse.

**Figure 1:**
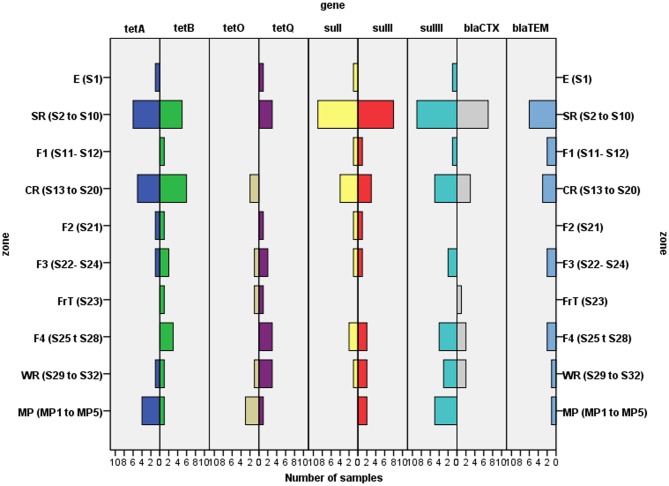
Detection of different antibiotic resistance genes (tetracycline, sulfonamide and beta-lactam genes) in different slaughterhouse zones and meat products.

**Figure 2:**
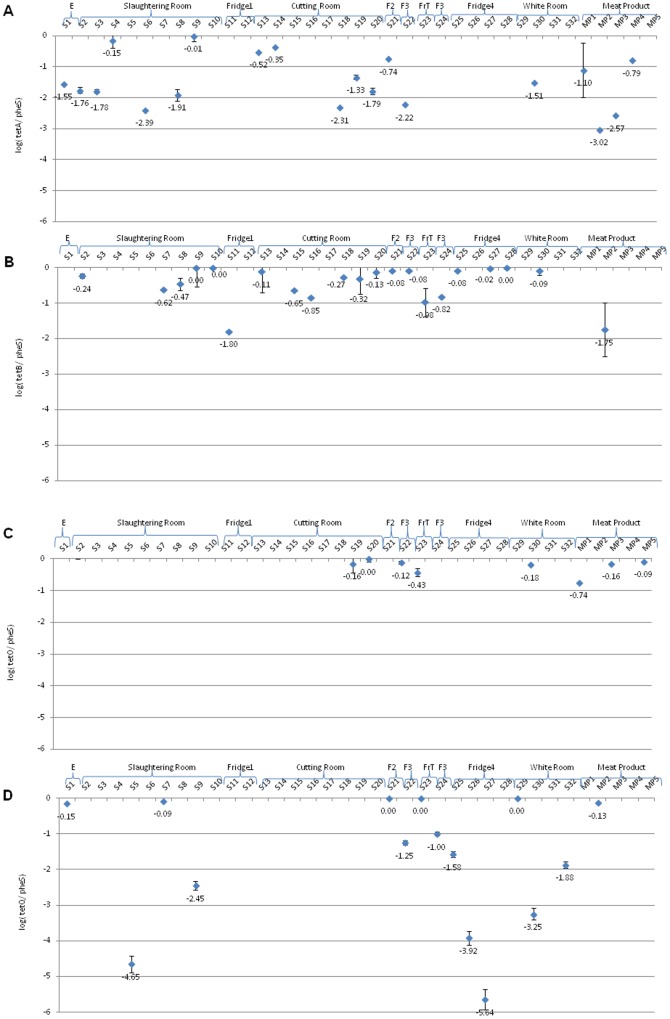
Relative concentrations of tetracyline resistance genes (A, *tetA*; B, *tetB*; C, *tetO*; D, *tetQ*) in different slaughterhouse zones and meat products.

**Figure 3:**
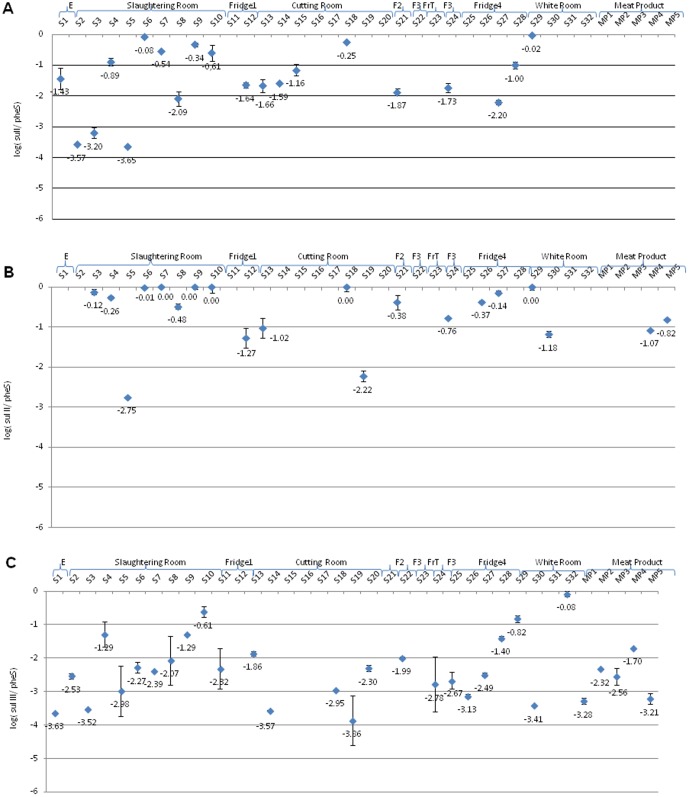
Relative concentrations of sulfonamide resistance genes (A, *sulI*; B, *sulII*; C, *sulIII*) in different slaughterhouse zones and meat products.

**Figure 4:**
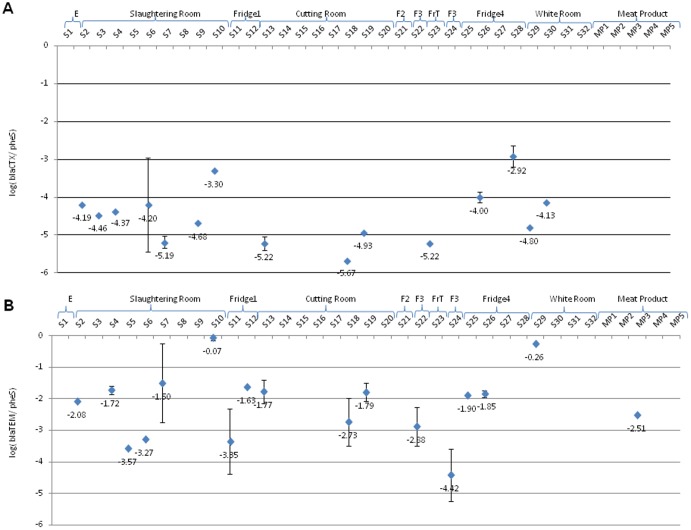
Relative concentrations of beta-lactam resistance genes (A, *bla_CTX_*; B, *bla_TEM_*) in different slaughterhouse zones and meat products.

Many slaughterhouse surfaces were found to harbor different ARG. PCR detected ARG in all zones (Fig. 1), with the entrance (E) and freezing tunnel (FrT) being zones having the least resistance diversity detected (four of nine determinants, 44%). Greatest diversities were found within the SR (slaughtering room) and CR (cutting room), where surfaces had 8/9 gene determinants. In terms of gene frequency on surfaces, the most widely distributed genes were *sulIII* (found on 25 surfaces, 78% of the total sampled) and *sulI*-*sulII*-*tetB* (18–20 surfaces, 56–63%), while gene *tetO* (only in 5 surfaces, 16%) was detected least frequently (Figs. 1–4). The *tetB* gene was the most prevalent being detected in 20 of the 32 surfaces analyzed (8 of 9 zones analyzed), followed by *tetA* and *tetQ* genes (14–15 samples of 6–7 zones) and *tetO* (8 samples of 4 zones) (Fig. 2); it, however, was not detected in the entrance (zone E) (Figs. 1, 2B). Concerning commercial meat products (MP), *sulIII* and *tetA* genes were detected in all or almost all samples, respectively, while *bla_CTX_* was absent in all meat samples analyzed (Figs. 1–4).

The distribution and broad presence of gene determinants creates great concern, considering the potential risks associated with the spread of ARG throughout meat chain production to end products. Although slaughtering and meat handling operations follow rigorously good hygienic practices, the risk of surface and end products contamination with ARB may occur.

**Figure 5:**
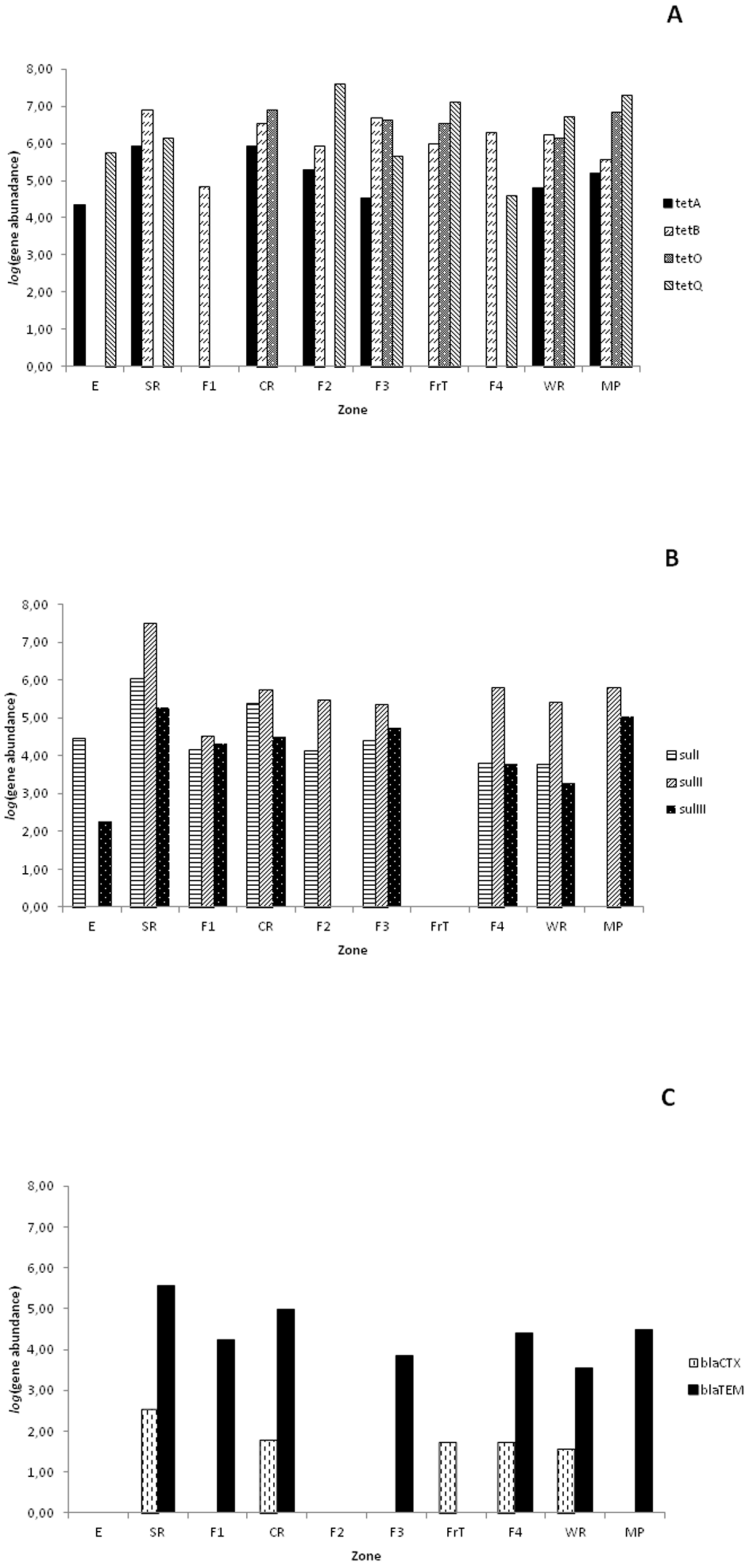
Gene abundance (absolute values per ml) of all antibiotic resistance genes (A, tetracyclines genes; B, sulfonamide genes; and C, beta-lactam genes) in different slaughterhouse zones and meat products.

To better understand the risks and the patterns of gene dissemination, measurements of genes were further evaluated quantitatively. Absolute abundances represent total genes swabbed per surface area (Figure 5). Highest abundances were obtained with tetracycline genes, mainly *tetB* and *tetQ* genes, which averaged (geometric mean) 10^5.5^ and 10^4.8^ genes/cm^2^ over the nine slaughterhouse zones, respectively (Fig. 5A). While *tetA* and *tetO* showed an average of 10^3.4^ and 10^2.9^ genes/cm^2^, respectively (Fig. 5A). Regarding *sul* gene abundances in slaughterhouse, *sulII* was the most abundant (10^4.4^ genes/cm^2^), followed by *sulI* (10^4^ genes/cm^2^) and *sulIII* (10^3.1^ genes/cm^2^) (Fig. 5B). Similarly, beta-lactam resistance gene *bla_TEM_* exhibited 10^3.0^ genes/cm^2^, while *bla_CTX_* was less abundant (10^1.0^ genes/cm^2^) (Fig. 5C). When analysis was done with commercial meat products, higher densities of all ARG except *bla_CTX_* were obtained (Fig. 5).

To approximate the percent resistance of total bacteria, ARG values were normalized by *pheS* values to provide relative abundances (Figures 2–4). Different determinants of the *tet* genes differed in prevalence among all slaughterhouse zones analyzed and even between samples recovered from surfaces within the same zone, thus strong spatial variations were detected depending on the ARG and location (Fig. 2). Relative concentrations of *tetB* gene were higher in almost all surfaces, oscillating between 10^−1.80^ and 10^0^ (genes/*pheS*) with gene densities more noticeable in SR, CR, F2, F3, F4 and WR (Fig. 2B). However, we only detected *tetB* gene in one sample of commercial meat products (MP2). Similarly, *tetA* gene was detected in all zones except F1, Frt and F4 zones (Figs. 1 and 2A). Relative concentrations of *tetA* gene were higher in both samples S4 and S9 of SR zone (approximately 10^0^ genes/*pheS*), followed by S13 and S14 of CR zone (10^−0.5^ and 10^−0.4^ genes/*pheS*), S21 of F2 zone and MP4 (about 10^−0.7^ and 10^−0.8^ genes/*pheS*, respectively) (Fig. 2A). Furthermore, we detected *tetA* gene in other surfaces (S1, S2, S3, S6, S8, S18, S19, S20, S22 and S30) belonging to different slaughterhouse zones (E, SR, CR, F3 and WR) and also meat products (MP1, MP2 and MP3) (ranging between 10^−3.0^ and 10^−1.1^ genes/*pheS*) (Fig. 2A).

Lavilla Lerma et al. [15] showed that the same goat and lamb slaughterhouse contained Gram negative bacteria (about 73% of which were *Pseudomonas* spp., *Escherichia coli* and non-identified Gram negative psychrotrophs), so the high prevalence of *tetA* and *tetB* genes (each encoding an efflux pump) was in agreement with those who reported tetracycline resistance in individual Gram negative bacteria isolated from animal foods and slaughterhouse environments [26]–[28]. Additionally the diversity of *tet* genes and their genetic mobility contribute significantly to their dissemination among many different bacteria [29].

Regarding tetracycline-resistance genes that encode ribosomal protection proteins (*tetO* and *tetQ*), they are commonly found in intestinal tracts of cattle and also in the environment; their quantification results were variable. *TetO* was only detected in CR, F3, FrT, WR and meat products (Fig. 2C) and the relative concentrations oscillated between 10^−0.74^ and 10^0^ (*tetO/pheS*) (Fig. 2C). However, *tetQ* gene was distributed throughout slaughterhouse surfaces except F1 and CR (Fig. 2D), being highly abundant in E (S1), SR (S7), F2 (S21), FrT (S23), WR (S29) and meat product (MP2) (*tetQ*/*pheS* were almost 10^0^), while in other surfaces (SR, F2, F3, F4 and WR) quantification of *tetQ* gene was variable (Fig. 2D).

The occurrence of *sul* genes varied and occurred in most slaughterhouse zones (Fig. 3A). Comparing relative concentrations of *sul* genes, higher abundances were found with *sulII* gene (10^−1.3^ and 10^0^ *sulII*/*pheS*) in most cases (Fig. 3B). However, *sulIII* gene was more disseminated throughout slaughterhouse surfaces being detected also in meat products (Fig. 3C). The high prevalence of sulfonamide resistance in animals and humans was largely reported all over the world due to the frequent use of those antimicrobials in veterinary, and their spread to humans via food chain was also documented [30], [31]. Furthermore, the genetic localization of *sul* genes on mobile elements may explain their wide distribution [32], [33].

Concerning beta-lactamases, relative concentrations of *bla_TEM_* gene was quantitatively higher (approximately 10^0^) than *bla_CTX_* and often detected in SR (S10) and WR (S29), and absent in E, F2 and FrT (Fig. 4). However, *bla_CTX_* was not detected in E, F1, F2, F3, FrT and meat products (Fig. 4A). The genes *bla_TEM_* and *bla_CTX_* are widespread among Gram negative pathogens [34]; however, in the present study despite the high prevalence of Gram negative bacteria in slaughterhouse environment, lower concentrations of beta-lactamase genes were detected in comparison with other genes.

### Analysis of total antibiotic throughout meat chain production

**Figure 6:**
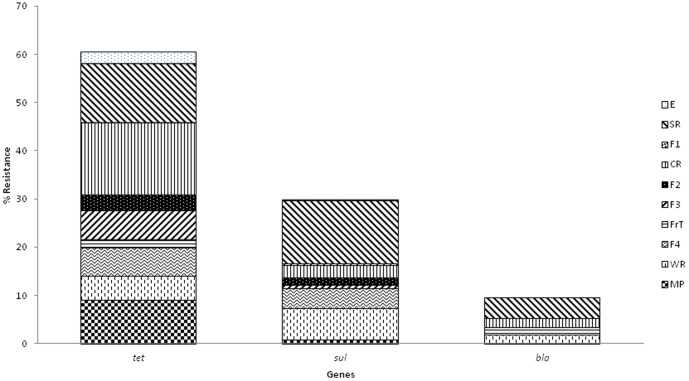
Percentage of tetracycline, sulfonamide and beta-lactam total resistances.

Analyses of resistance genes provide quantitative information for risk assessment in each slaughterhouse zone and also meat products. Analysis of the sum of measured tetracycline resistance genes (*tetA*, *tetB*, *tetO* and *tetQ*), sulfonamide resistance genes (*sulI*, *sulII* and *sulIII*) and beta-lactam resistance genes (*bla_TEM_* and *bla_CTX_*) in different slaughterhouse zones and meat products showed that tetracycline genes were most prevalent in goat and lamb slaughterhouse zones and meat products (about two and ten orders of magnitude higher than sulfonamide and beta-lactamase groups, respectively) (Fig. 6). This data is not surprising since tetracycline genes are often spread over many promiscuous conjugative genetic elements [29], [35], thus detection of *tet* genes was possible in a diversity of environmental bacteria (in soil, sludge, wastewater, river water, and agriculture) [36]–[40] and foods [41]. The most important reservoir of tetracycline genes were CR, SR and MP, while sulfonamide and beta lactamase groups were mainly observed in SR (Fig. 6).

### Statistical analysis

Statistical analysis of the relative concentrations of each ARG showed that the differences were not significant (*p*>0.05) between slaughterhouse zones and meat products concerning *tetA*, *tetO*, *tetQ*, *sulI*, *sulII* and *sulIII* genes (Table 2). However, the differences in the relative concentrations of *tetB*, *bla_CTX_* and *bla_TEM_* genes were significant (*p*<0.05) among slaughterhouse zones and also meat products (Table 2). The data suggests that resistance could be acquired by a spread of genetic trait through slaughterhouse zones and also meat products; the exception would be *tetB*, *bla_CTX_* and *bla_TEM_* genes, which appear as ‘hot spots’ within the meat processing stream. ARG may migrate with ARB throughout slaughterhouse zones. Besides indirect transmission of resistant bacteria through the food chain by consumption of animal foods, resistance acquisition can occur by direct contact of farms and slaughterhouse workers and veterinarians, which can be other vectors by which ARB are spread to the community and the environment [42].

**Table 2:** Quantification of ARGs in different slaughterhouse zones and end products.

Gene	Zone	Mean ± SD Stat Signif Diff*	Gene	Zone	Mean ± SD Stat Signif Diff*
			***sulI***	F2	−1.875±0.007 A
				F3	−1.730±0.004 A
***tetA***	F3	−2.219±0.019 A		SR	−1.662±1.473 A
	MP	−1.870±1.091 A		F1	−1.641±0.049 A
	E	−1.550±0.029 A		F4	−1.600±0.845 A
	WR	−1.514±0.012 A		E	−1.430±0.055 A
	SR	−1.334±1.000 A		CR	−1.165±0.651 A
	CR	−1.260±0.831 A		WR	−0.024±0.002 A
	F2	−0.743±0.033 A	***sulII***	F1	−1.270±0.055 A
***tetB***				CR	−1.081±1.112 A
	F1	−1.797±0.040 A		MP	−0.946±0.181 A
	MP	−1.750±0.040 A		WR	−0.786±0.680 A
	FrT	−0.975±0.032 AB		F3	−0.764±0.048 A
	F3	−0.450±0.523 BC		SR	−0.500±0.932 A
	CR	−0.389±0.297 BC		F2	−0.380±0.026 A
	SR	−0.184±0.318 BC		F4	−0.253±0.162 A
	WR	−0.090±0.019 C	***sulIII***	E	−3.629±0.060 A
	F2	−0.083±0.005 C		CR	−2.910±0.839 A
	F4	−0.034±0.042 C		MP	−2.613±0.657 A
				F4	−2.425±0.732 A
				F3	−2.386±0.555 A
***tetO***	FrT	−0.428±0.017 A		F1	−2.318±0.044 A
	MP	−0.330±0.361 A		SR	−2.104±0.910 A
	WR	−0.184±0.014 A		WR	−1.437±1.752 A
	F3	−0.123±0.036 A	***bla_CTX_***	CR	−5.276±0.374 A
	CR	−0.060±0.139 A		FrT	−5.218±0.025A
				WR	−4.467±0.472 AB
***tetQ***	F4	−3.713±2.039 A		SR	−4.342±0.574 AB
				F4	−3.459±0.765 B
	SR	−2.397±2.284 A	***bla_TEM_***	F3	−3.652±1.087 A
	WR	−1.709±1.632 A		MP	−2.512±0.055 AB
	F3	−1.129±0.178 A		F1	−2.490±1.675 AB
	E	−0.152±0.033 A		CR	−2.095±0.547 AB
	MP	−0.128±0.019 A		SR	−2.036±1.275 AB
	F2	0.008±0.030 A		F4	−1.873±0.038 AB
	FrT	0.004±0.002 A		WR	−0.257±0.022 B

*Different letters represent significant differences according to Tukey or Games- Howell tests (*p*<0.05).

Significant positive correlations between different sampling zones/meat products and the total relative concentrations of ARG were observed in CR and F2, SR or WR; SR and F1 or WR; F1 and F2; MP and F4, while negative correlations were only detected between F1 and F3 (Table 3). Furthermore, *tetQ* positively correlated with *tetO* and *sulI*, however, *tetA* gene negatively correlated with *bla_TEM_* gene (Table 4). In all cases, correlations of ARGs were highly significant (*p*<0.01 or *p*<0.05). Moreover, a broad relationship between some slaughterhouse zones (mainly between SR, CR and WR) throughout meat chain production indicated flow of resistance genes by handling, carcasses, transport and utensils.

**Table 3:** Correlations between the relative concentrations of ARGs in different slaughterhouse zones (per *pheS* gene; *log*-transformed).

Zone	Entrance	Slaughtering Room	Fridge 1	Cutting Room	Fridge 2	Fridge 3	Freezing Tunnel	Fridge 4	White Room	Meat Products
**Entrance**	1									
**Slaughtering Room**	0.052	1								
**Fridge1**	0.427	0.672*	1							
**Cutting Room**	−0.234	0.735**	0.480	1						
**Fridge2**	0.348	0.213	0.712*	0.795*	1					
**Fridge3**	0.082	0.029	−0.691*	0.056	−0.268	1				
**Freezing Tunnel**	0.332	0.025	0.296	0.180	0.190	0.310	1			
**Fridge 4**	0.150	−0.114	−0.201	−0.060	0.374	0.301	0.405	1		
**White Room**	0.118	0.647**	0.084	0.609**	0.078	0.493	0.223	0.050	1	
**Meat Products**	−0.069	0.132	−0.337	0.289	−0.288	−0.008	0.423	0.608**	−0.064	1

*Asterisk denoted significant correlation at *p*<0.05 level (2- tailed).

**Double asterisk denoted significant correlation at *p*<0.01 level (2- tailed).

**Table 4:** Correlations between relative concentrations of different ARGs (per *pheS* gene; *log*-transformed).

Gene	*bla_CTX_*	*bla_TEM_*	*tetA*	*tetB*	*tetO*	*tetQ*	*sulI*	*sulII*	*sulIII*
***bLa_CTX_***	1								
***bLA_Tem_***	−0.318	1							
***tetA***	0.212	−0.514*	1						
***tetB***	0.138	−0.213	−0.024	1					
***tetO***	−0.220	−0.135	−0.237	0.148	1				
***tetQ***	−0.126	0.168	−0.385	−0.007	0.641*	1			
***sulI***	−0.152	−0.373	0.039	0.187	0.513	0.535*	1		
***sulII***	−0.224	0.134	−0.128	0.348	−0.025	0.295	0.336	1	
***sulIII***	−0.380	−0.316	0.012	−0.010	0.245	−0.054	0.234	0.065	1

*Asteristik denoted significant correlation at *p*<0.05 level (2- tailed).

## Conclusions

Slaughterhouse surfaces and meat products act as large reservoirs of ARG especially *tet* genes. The greatest risk appears to be located in cutting room (CR) and slaughtering room (SR), with evidence of ARG (and ARB) ending up in end products (MP). These data should importantly be considered to reduce the risk of gene transfer throughout slaughterhouse zones. Furthermore, total resistance in SR, CR and WR zones, and also F4 and MP strongly correlated, suggesting resistance disseminated throughout slaughterhouse zones by carry-over contamination; control of those key zones in slaughterhouse (SR, CR and WR) would be a good strategy to reduce the risks of transmission and avoid food-safety problems with food safety by adequate disinfection methods.

## References

[1] Wegener HC (2003) Antibiotics in animal feed and their role in resistance development. Curr Opin Microbiol 6:439–445. 14572534 10.1016/j.mib.2003.09.009

[2] Barza MD, Gorbach SL (2002) The need to improve antimicrobial use in agriculture: ecological and human health consequences. Clin Infect Dis 34:S71–144.11988874

[3] Fey PD, Safranek TJ, Rupp ME, Dunne EF, Ribot E, et al (2000) Ceftriaxone-resistant *Salmonella* infection acquired by a child from cattle. N Engl J Med 342:1242–1249. 10781620 10.1056/NEJM200004273421703

[4] Holmberg SD, Osterholm MT, Senger KA, Cohen ML (1984) Drug-resistant *Salmonella* from animals fed antimicrobials. N Engl J Med 311:617–622. 6382001 10.1056/NEJM198409063111001

[5] Hummel R, Tschape H, Witte W (1986) Spread of plasmid-mediated nourseothricin resistance due to antibiotic use in animal husbandry. J Basic Microbiol 26:461–466. 3033194 10.1002/jobm.3620260806

[6] Levy SB, FitzGerald GB, Macone AB (1976) Changes in intestinal flora of farm personnel after introduction of a tetracycline-supplemented feed on a farm. N Engl J Med 295:583–588. 950974 10.1056/NEJM197609092951103

[7] Marshall BM, Levy SB (2011) Food Animals and Antimicrobials: Impacts on Human Health. Clin Microbiol Rev 24:718–733. 21976606 10.1128/CMR.00002-11 PMC3194830

[8] Aarestrup FM (2005) Veterinary drug usage and antimicrobial resistance in bacteria of animal origin. Basic Clin Pharmacol 96:271–81. 10.1111/j.1742-7843.2005.pto960401.x 15755309

[9] Schjørring S, Krogfelt KA (2011) Assessment of bacterial antibiotic resistance transfer in the gut. Int J Microbiol 2011:1–10. 10.1155/2011/312956 PMC3034945 21318188

[10] Stecher B, Denzler R, Maier L, Bernet F, Sanders MJ, et al (2012) Gut inflammation can boost horizontal gene transfer between pathogenic and commensal *Enterobacteriaceae* . Proc Natl Acad Sci USA 109:1269–1274. 22232693 10.1073/pnas.1113246109 PMC3268327

[11] Brabban AD, Hite E, Callaway TR (2005) Evolution of foodborne pathogens via temperate bacteriophage-mediated gene transfer. Foodborne Pathog Dis 2:287–303. 16366852 10.1089/fpd.2005.2.287

[12] Chopra I, Roberts M (2001) Tetracycline Antibiotics: Mode of Action, Applications, Molecular Biology, and Epidemiology of Bacterial Resistance. Microbiol Mol Biol Rev 65:232–260. 11381101 10.1128/MMBR.65.2.232-260.2001 PMC99026

[13] Sunde M, Norstrom M (2006) The prevalence of, associations between and conjugal transfer of antibiotic resistance genes in *Escherichia coli* isolated from Norwegian meat and meat products. J Antimicrob Chemother 58:741–7. 16931539 10.1093/jac/dkl294

[14] Smith DeWaal C, Vaughn Grooters S (2013) Antibiotic resistance in foodborne pathogens. Washington, DC: Center for Science in the Public Interest. pp. 1–22.

[15] Lavilla Lerma L, Benomar N, Gálvez A, Abriouel H (2013) Prevalence of bacteria resistant to antibiotics and/or biocides on meat processing plant surfaces throughout meat chain production. Int J Food Microbiol 161:97–106. 23279818 10.1016/j.ijfoodmicro.2012.11.028

[16] De los Reyes-Gavilan CG, Limsowtin GKY, Tailliez P, Séchaud L, Acholas JP (1992) A *Lactobacillus helveticus*-specific DNA probe detects restriction fragment length polymorphisms in this species. Appl Environ Microbiol 58:3429–3432. 16348794 10.1128/aem.58.10.3429-3432.1992 PMC183119

[17] Sambrook J, Fritsch EF, Maniatis T (1989) Molecular Cloning: A Laboratory Manual. Cold Spring Harbor laboratory press. Cold Spring Harbor.

[18] Smith MS, Yang RK, Knapp CW, Niu Y, Peak N, et al (2004) Quantification of Tetracycline Resistance Genes in Feedlot Lagoons by Real-Time PCR. Appl Environ Microbiol 70:7372–7377. 15574938 10.1128/AEM.70.12.7372-7377.2004 PMC535139

[19] Naser SM, Thompson FL, Hoste B, Gevers D, Dawyndt P, et al (2005) Application of multilocus sequence analysis (MLSA) for rapid identification of *Enterococcus* species based on *rpoA* and *pheS* genes. Microbiol 151:2141–2150. 10.1099/mic.0.27840-0 16000705

[20] Dancey C, Reidy J (2004). Statistics without Maths for Psychology: using SPSS for Windows. London: Prentice Hall.

[21] Aarestrup FM, Wegener HC, Collignon P (2008) Resistance in bacteria of the food chain: Epidemiology and control strategies. Expert Rev Anti Infect Ther 6:733–750. 18847409 10.1586/14787210.6.5.733

[22] Guardabassi L, Stegger M, Skov R (2007) Retrospective detection of methicillin resistant and susceptible *Staphylococcus aureus* ST398 in Danish slaughter pigs. Vet Microbiol 122:384–386. 17467199 10.1016/j.vetmic.2007.03.021

[23] Van den Bogaard A, London N, Driessen C, Stobberingh E (2001) Antibiotic resistance of faecal ***Escherichia coli*** in poultry, poultry farmers and poultry slaughterers. J Antimicrob Chemoth 47:763–71. 10.1093/jac/47.6.763 11389108

[24] Gregova G, Kmetova M, Kmet V, Venglovsky J, Feher A (2012) Antibiotic resistance of *Escherichia coli* isolated from a poultry slaughterhouse. Ann Agric Environ Med 19:75–77. 22462449

[25] Brtkova A, Bujdakova H (2009) Antibiotic resistance in *Enterococcus* isolates from poultry swabs in Slovakia. J Food Nutr Res 48:121–128.

[26] Aradhye AA, Kolhe RP, Bhong CD, Deshpande PD, Lokhande SD, et al (2014) Prevalence of antimicrobial resistant pathotypes of *Escherichia coli* in beef cattle and slaughterhouse premise. Afr J Microbiol Res. 8:277–286.

[27] Gow SP, Walder CL, Harel J, Boerlin P (2008) Associations between antimicrobial resistance genes in fecal generic *Escherichia coli* isolates from cow-calf herds in western Canada. Appl Environ Microbiol 74:3658–3666. 18424533 10.1128/AEM.02505-07 PMC2446549

[28] Skočková A, Cupáková S, Karpíšková R, Janštová B (2012) Detection of tetracycline resistance genes in *Escherichia coli* from raw cow's milk. J Microbiol Biotech Food Sci 1:777–784.

[29] Roberts MC (2005) Update on acquired tetracycline resistance genes. FEMS Microbiol Lett 245:195–203. 15837373 10.1016/j.femsle.2005.02.034

[30] Hammerum AM, Sandvang D, Andersen SR, Seyfarth AM, Porsbo LJ, et al (2006) Detection of sul1, sul2 and sul3 in sulphonamide resistant ***Escherichia coli*** isolates obtained from healthy humans, pork and pigs in Denmark. Int J Food Microbiol 106:235–237. 16216373 10.1016/j.ijfoodmicro.2005.06.023

[31] Trobos M, Jakobsen L, Olsen KE, Frimodt-Moller N, Hammerum AM, et al (2008) Prevalence of sulphonamide resistance and class 1 integron genes in ***Escherichia coli*** isolates obtained from broilers, broiler meat, healthy humans and urinary infections in Denmark. Int J Antimicrob Agents 32:367–369. 18583102 10.1016/j.ijantimicag.2008.04.021

[32] Antunes P, Machado J, Sousa JC, Peixe L (2005) Dissemination of sulfonamide resistance genes (sul1, sul2, and sul3) in Portuguese ***Salmonella enterica*** strains and relation with integrons. Antimicrob Agents Chemother 49:836–839. 15673783 10.1128/AAC.49.2.836-839.2005 PMC547296

[33] Bean DC, Livermore DM, Hall LM (2009) Plasmids imparting sulfonamide resistance in ***Escherichia coli***: implications for persistence. Antimicrob Agents Chemother 53:1088–1093. 19075061 10.1128/AAC.00800-08 PMC2650533

[34] Coque TM, Novais A, Carattoli A, Poirel L, Pitout J, et al (2008) Dissemination of clonally related *Escherichia coli* strains expressing extended-spectrum _-lactamase CTX-M-15. Emerg. Infect. Dis. 14:195–200. 10.3201/eid1402.070350 PMC2600198 18258110

[35] Thaker M, Spanogiannopoulos P, Wright GD (2010) The tetracycline resistome. Cell Mol Life Sci 67:419–431. 19862477 10.1007/s00018-009-0172-6 PMC11115633

[36] Auerbach EA, Seyfried EE, McMahon KD (2007) Tetracycline resistance genes in activated sludge wastewater treatment plants. Water Res 41: 1 143–1151. 10.1016/j.watres.2006.11.045 17239919

[37] Pei R, Kim SC, Carlson KH, Pruden A (2006) Effect of river landscape on the sediment concentrations of antibiotics and corresponding antibiotic resistance genes (ARG). Water Res 40:2427–2435. 16753197 10.1016/j.watres.2006.04.017

[38] Knapp CW, Dolfing J, Ehlert PAI, Graham DW (2010) Evidence of increasing antibiotic resistance gene abundances in archived soils since 1940. Environ Sci Technol 44:580–587. 20025282 10.1021/es901221x

[39] Zhang XX, Zhang T (2011) Occurrence, abundance, and diversity of tetracycline resistance genes in 15 sewage treatment plants across China and other global locations. Environ Sci Technol 45:2598–2604. 21388174 10.1021/es103672x

[40] Zhu YG, Johnson TA, Su JQ, Qiao M, Guo GX, et al (2013) Diverse and abundant antibiotic resistance genes in Chinese swine farms. Proc Natl Acad Sci USA 110:3435–3440. 23401528 10.1073/pnas.1222743110 PMC3587239

[41] Florez AB, Ammor MS, Mayo B (2008) Identification of *tet*(M) in two *Lactococcus lactis* strains isolated from a Spanish traditional starter-free cheese made of raw milk and conjugative transfer of tetracycline resistance to lactococci and enterococci. Int J Food Microbiol 121:189–194. 18068255 10.1016/j.ijfoodmicro.2007.11.029

[42] Molbak K, Baggesen DL, Aarestrup FM, Ebbesen JM, Engberg J, et al (1999) An outbreak of multidrug-resistant, quinolone- resistant *Salmonella enterica* serotype Typhimurium DT104. N Engl J Med 341:1420–1425. 10547404 10.1056/NEJM199911043411902

[43] Ng LK, Martin I, Alfo M, Mulvey M (2001) Multiplex PCR for the detection of tetracycline resistance genes. Mol Cell Probes 15:209–215. 11513555 10.1006/mcpr.2001.0363

[44] Birkett CI, Ludlam HA, Woodford N, Brown DF, Brown NM, et al (2007) Real-time TaqMan PCR for rapid detection and typing of genes encoding CTX-M extended-spectrum β-lactamases. J Med Microbiol 56:52–55. 17172517 10.1099/jmm.0.46909-0

